# Sclerodermiform Dermatitis After Coronary Artery Bypass Graft With an Internal Thoracic Artery Conduit

**DOI:** 10.7759/cureus.19721

**Published:** 2021-11-18

**Authors:** Caroline A Gerhardt, Stefanie Grewe, Nora Vera, Alexander Reese, Brooke Baldwin

**Affiliations:** 1 Dermatology, University of South Florida Morsani College of Medicine, Tampa, USA; 2 Pathology, University of South Florida, Tampa, USA; 3 Dermatology and Cutaneous Surgery, University of South Florida Morsani College of Medicine, Tampa, USA; 4 Pathology and Laboratory Medicine, University of South Florida Morsani College of Medicine, Tampa, USA; 5 Dermatology, James A. Haley Veterans' Hospital, Tampa, USA

**Keywords:** internal thoracic artery harvesting site, sclerodermatous, indurated plaque, cabg, sclerodermiform dermatitis

## Abstract

Coronary artery bypass grafting is a common surgical procedure that often uses the saphenous vein, internal thoracic artery, or radial artery as a conduit to improve blood circulation to the heart. When a blockage or impediment to arterial flow is noted, this procedure is undertaken to ensure the myocardium receives the blood it needs to function optimally. Infrequently, dermatoses overlying the conduit site may be observed, notably with the saphenous vein harvest site. Here we report the first case of sclerodermiform dermatitis occurring at the internal thoracic artery donor graft site. This unique case is important for providers to be aware of when evaluating a patient post-operatively who presents with new-onset dermatologic changes at the site of previous donor harvesting to ensure optimal treatment and management.

## Introduction

Coronary artery bypass grafting (CABG) is the gold-standard treatment for multi-vessel coronary artery disease. This procedure is the most common cardiac surgery performed worldwide [1], with more than 200,000 CABG procedures performed in the United States annually [2].

The most frequently used conduits for CABG are the saphenous vein, internal thoracic artery, and radial artery [1]. Initially, saphenous veins were the most widely utilized conduits for CABG procedures due to their increased length and easy accessibility. As the procedure was developed over time, it was determined the internal thoracic artery was superior due to the decreased incidence of atherosclerotic and hyperplastic changes that result in long-term improved patient outcomes because of its excellent long-term patency [1]. The radial artery has recently emerged as a popular conduit option because of its accessibility for harvesting and ease of anastomosing, though it exhibits vulnerability to vasospasm [1]. Conduit sites are selected based on various factors, such as harvest location, notably due to the difference in elasticity, rate of atherosclerotic development, intimal hyperplasia predilections, and vasospasm propensity [1]. As with any surgical procedure, the risk of deep wound infection exists and should be communicated to patients when deciding on the best surgical plan.

Dermatoses following CABG are rare complications that have been reported in the literature when the saphenous vein is used for harvesting. Previously cited dermatoses following saphenous vein harvesting include ecchymoses, telangiectasias, eczematous dermatitis, xerosis, hypertrophic scarring, cellulitis, and cutaneous ulceration [3,4]. Of note, sclerodermiform dermatitis has been reported in a limited number of patients who underwent saphenous vein stripping for varicose veins [5]. Cutaneous manifestations after harvesting have not been reported in the literature utilizing the internal thoracic artery as a conduit. Given the superior outcomes noted with this artery harvest site, clinicians must recognize and diagnose these potential manifestations that can occur after CABG at this site on the chest to avoid potential misdiagnosis. 

## Case presentation

**Figure 1:**
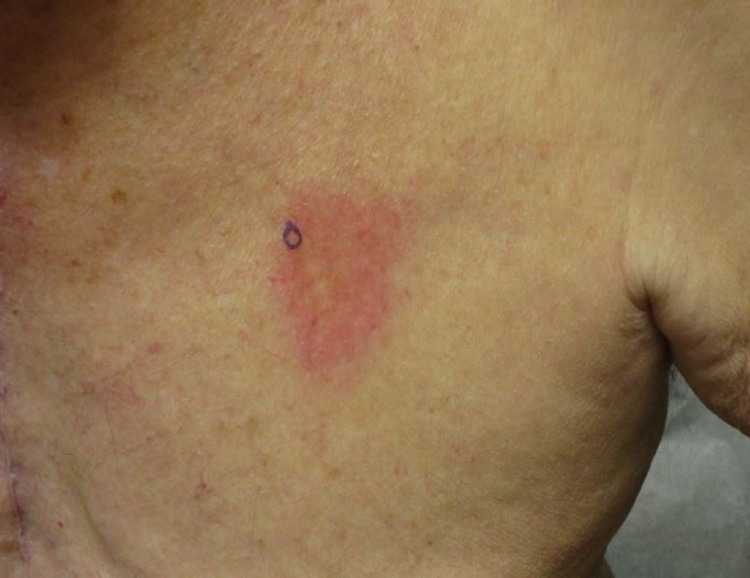
Well-demarcated erythematous, indurated plaque overlying the harvest site of the left internal thoracic artery.

The patient is an 81-year-old male with medical comorbidities of hypertension, coronary artery disease, and non-melanoma skin cancer who presented with a new cutaneous manifestation. He reported a two-week history of a progressively enlarging asymptomatic lesion on his left chest. Five months prior he had a CABG procedure with internal thoracic artery harvesting without the use of fluoroscopy. Physical examination revealed a well-demarcated erythematous, indurated plaque overlying the previous harvest site of his left internal thoracic artery (Figure 1).

**Figure 2:**
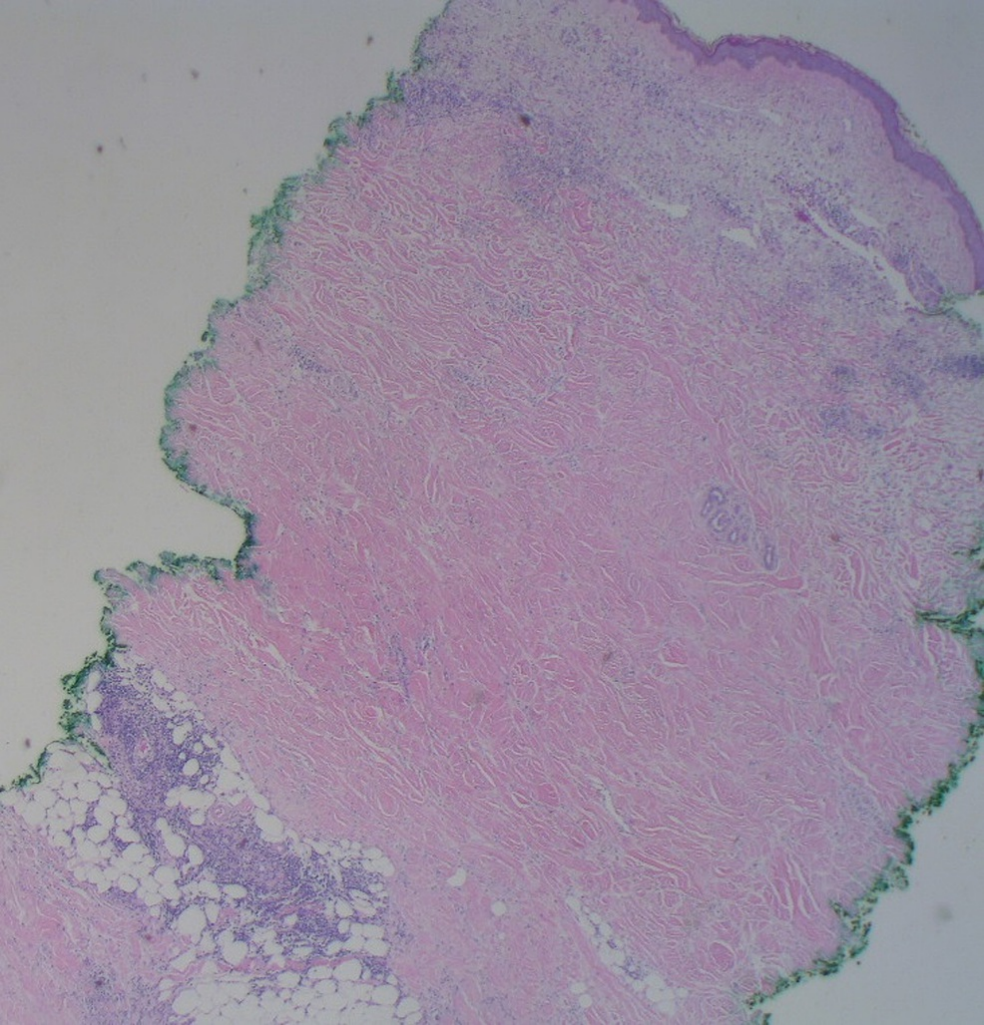
Diffuse dermal sclerosis and underlying panniculitis (H&E, 20X). H&E: Hematoxylin and eosin stain

**Figure 3:**
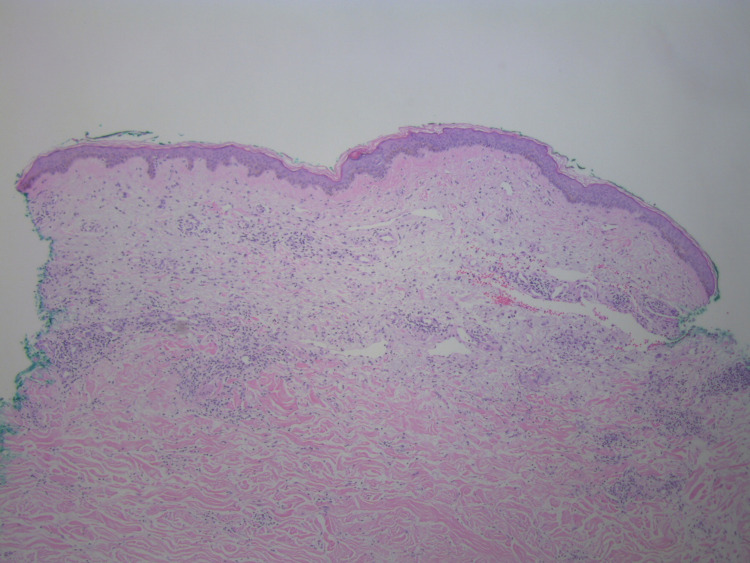
The epidermis is uninvolved; mild fibrosis and a perivascular inflammatory infiltrate is seen in the superficial dermis (H&E, 40X). H&E: Hematoxylin and eosin stain

**Figure 4:**
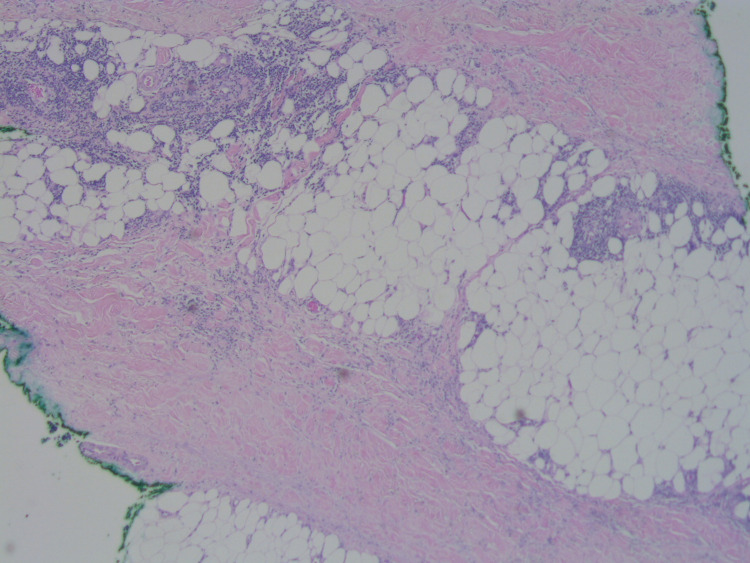
Septal panniculitis (H&E, 40x). H&E: Hematoxylin and eosin stain

A punch biopsy was performed to further classify this new development; histopathologic evaluation revealed an uninvolved epidermis with diffuse dermal sclerosis and underlying panniculitis (Figure 2, Figure 3, Figure 4). 

In the absence of any prior history of scleroderma (cutaneous or systemic), these localized sclerodermatous changes were attributed to recent arterectomy. Given the asymptomatic nature of the plaque, the management options were discussed with the patient, and he opted for observation during his bi-annual skin examinations.

## Discussion

Cutaneous manifestations have previously been reported at other sites of conduit recovery; however, to our knowledge, this is the first reported case of sclerodermiform dermatitis involving the internal thoracic artery harvesting site.

In 1999, French et al. reported three cases of sclerodermiform dermatitis following saphenous vein stripping for varicose veins [5]. These cases presented as hypopigmented and indurated linear plaques following the distribution of the stripped vein, presenting between two to six years after their procedures. Biopsy of the lesions demonstrated epidermal atrophy, diffuse dermal sclerosis, perivascular and interstitial lymphocytic infiltrate, and deep infiltrates in the septa and adipose lobules. The biopsy of our patient had similar histopathologic findings to those reported in this paper. However, unlike the previously reported cases with a linear distribution following the stripped saphenous vein, the cutaneous findings in our case were patch-like and limited to the area surrounding the scar.

The pathophysiology underlying these changes is unknown. It is hypothesized that damage to the nerves, vasculature, and lymphatics leads to changes in the skin and subcutaneous tissue. One proposed mechanism for the sclerodermatous changes includes local hypoxia as the stimulus for transforming growth factor-beta upregulation and collagen synthesis [5,6]. This suggests that decreased oxygenation from disruption of the vasculature may favor molecular processes that lead to localized sclerosis. One study demonstrated reduced sternal and parasternal skin perfusion on laser doppler imaging following internal thoracic artery harvesting [7]. This finding further supports local skin and subcutaneous hypoxia as a possible mechanism for the sclerodermatous changes seen in our patient. 

Distinct from the sclerodermatous changes seen post saphenous vein removal is eczematous changes. These changes have been reported more frequently than sclerodermatous changes. Since 1981, less than 20 reported cases have been reported in the literature [3,4,8-11]. The cases have presented as eczematous dermatitis in a linear distribution along the venectomy scar approximately one to nine months following saphenous vein harvesting for CABG. Patients commonly reported neurosensory deficits along the same distribution, and some also had pruritis. Biopsy of these lesions demonstrated spongiotic dermatitis with perivascular infiltrates of mononuclear cells in the dermis. Most of the patients improved with a topical steroid with medium potency.

There is also an interesting association between cutaneous changes and neurosensory deficits. Hruza et al. observed the synchronous resolution of neuropathy and dermatitis in the two reported cases of eczematous dermatitis following CABG with saphenous vein grafting [9]. The proposed mechanisms for these findings are denervation causing motor, sensory, and autonomic distraction resulting in xerosis, loss of sweating, loss of sensation, loss of barrier function predisposing to dermatitis [4].

More recently, the first prospective study looking at cutaneous findings following CABG with saphenous vein grafting was reported in 2012 [10]. This study demonstrated that cutaneous findings at the donor graft site occurred in 60% of their patients. The most common symptom was anesthesia at the site (49/100), and the most common cutaneous finding was xerosis (17/100). They also reported six cases of neuropathy dermatitis, with neuropathy occurring in the same distribution as dermatitis along the distribution of the saphenous vein.

Neurosensory and vascular disruptions following donor graft removal can lead to cutaneous changes. Dermatologists and cardiothoracic surgeons should be aware of these findings so that they can adequately diagnose, treat, and counsel patients.

## Conclusions

Dermatoses following saphenous vein harvesting are widely documented. We present this case because it is the first reported case of sclerodermiform dermatitis at the internal thoracic artery graft site. We hope to bring awareness to this potential complication following CABG and graft site removal.
